# Autonomous Oscillation of Polymer Chains Induced by the Belousov–Zhabotinsky Reaction

**DOI:** 10.3390/s140101497

**Published:** 2014-01-15

**Authors:** Yusuke Hara, Yoshiko Takenaka

**Affiliations:** Nanosystem Research Institute, NRI, National Institute of Advanced Science and Technology, AIST, Central 5-2, 1-1-1 Higashi, Tsukuba 305-8565, Japan; E-Mail: takenaka.yoshiko@aist.go.jp

**Keywords:** self-oscillation, polymer chain, BZ reaction, molecular robot

## Abstract

We investigated the self-oscillating behaviors of two types of polymer chains induced by the Belousov–Zhabotinsky (BZ) reaction. One consisted of *N*-isopropylacrylamide (NIPAAm) and the Ru catalyst of the BZ reaction, and the other consisted of NIPAAm, the Ru catalyst, and acrylamide-2-methylpropanesulfonic acid (AMPS) with a negatively charged domain as a solubility control site. A comparison of the two types of self-oscillation systems showed that the anionic AMPS portion of the polymer chain significantly affected the self-oscillating behavior under strongly acidic condition. The periods of self-oscillation for the two types of self-oscillating polymer chains were investigated by changing the initial concentrations of the three BZ substrates and the temperature. As a result, it was demonstrated that the period of self-oscillation could be controlled by the concentration of the BZ substrates and the temperature. Furthermore, the activation energies of the two types of the self-oscillating polymer chains gave similar values as normal BZ reactions, *i.e.*, not including the self-oscillating polymer system with a Ru moiety. In addition, it was clarified the activation energy was hardly affected by the initial concentration of the three BZ substrates.

## 1. Introduction

There have been many reports on nanodevices and molecular machines based on DNA, proteins, and polymers [1–5]. Moreover, molecular devices fabricated using DNA base sequences have generated significant interest because DNA can be used for molecular programming [6–11]. DNA base sequences can be used to design two-dimensional and three-dimensional DNA nanostructures in solution. DNA structures are designed using a rigid motif including several DNA junctions and building blocks. However, DNA nanostructures are too rigid to drive dynamically. Therefore, stimuli-responsive polymer-based materials have been investigated for the fabrication of molecular devices and molecular machines [12–16]. The properties and functions of stimuli-responsive polymeric materials can be altered by external stimuli. Recently, thermoresponsive poly(*N*-isopropylacrylamide) (PNIPAAm) has been investigated, especially for use in microfluidic devices [17,18]. In order to drive stimuli-responsive polymer materials, external devices for controlling the external stimuli are needed.

In contrast, organic systems can generate autonomous motion without external stimuli. In order to produce autonomous molecular machines resembling living organisms, self-oscillating polymeric materials have been developed and investigated [19–21]. The energy source in these self-oscillating polymer materials is the Belousov–Zhabotinsky (BZ) reaction. The BZ reaction is a well-known oscillating reaction that is accompanied by spontaneous redox oscillations to generate a wide variety of nonlinear phenomena [22–27]. The overall process of the BZ reaction is the oxidation of an organic substrate by an oxidizing agent in the presence of a catalyst under strongly acidic conditions. In the BZ reaction, changes in the oxidation state of ruthenium tris(2,2′-bipyridine), the metal catalyst in the BZ reaction, occur periodically. As the oxidation state of the Ru catalyst changes, the solubility of the Ru catalyst changes simultaneously. In previous studies, polymer chains covalently bonded to the Ru catalyst were synthesized to convert the chemical energy to the driving force for the polymer chain oscillations [21]. As the oxidation state of the Ru catalyst moiety changes in the BZ reaction, the solubility of the polymer chain changes concurrently. As a result, the self-oscillating polymer chains undergo aggregation and disaggregation upon self-oscillation induced by the BZ reaction under constant temperature conditions. In previous investigations, Hara *et al.* developed self-oscillating polymer chains with acrylamide-2-methylpropane sulfonic acid (AMPS) [28]. The AMPS-containing polymer chains could control the self-oscillation and cause the viscosity self-oscillation under the acid-free conditions [29,30]. However, the influence of the concentrations of the three BZ substrates and the effect of temperature on the self-oscillating behavior (waveform and period) were not clarified, especially in regard to comparison with the conventional self-oscillating polymer chains (poly(NIPAAm-*co*-Ru(bpy)_3_) under strongly acidic conditions. This detailed information about the self-oscillating polymer chains is significantly important in the design of novel autonomous molecular robots.

Here, we investigate the influence of the anionic domain in the polymer chain on the self-oscillating behavior by utilizing two types of polymer chains: one system consisted of NIPAAm and the BZ Ru catalyst, *i.e.*, poly(NIPAAm-*co*-Ru(bpy)_3_), and the other consisted of NIPAAm, the Ru catalyst, and negatively charged AMPS as a solubility control site, *i.e.*, poly(NIPAAm-*co*-Ru(bpy)_3_-*co*-AMPS). By using two types of the self-oscillating polymer chain, we studied the effect of the anionic domain on the Lower Critical Solution Temperature (LCST) and the self-oscillating behaviors under strong acidic conditions. In addition, the influences of the initial concentrations of the BZ reaction substrates and the temperature on the period of the two types of the self-oscillating polymer chains were clarified. Some results (Figures 6 and 7) were already included in a previous report [31]. However, that report was not peer reviewed and the number of pages allowed was limited. Therefore, in this paper, in order to clarify the self-oscillating behaviors of the two types of the polymer chains in greater detail, we provide more information (self-oscillating behaviors, LCST and the relationship between temperature and period) and further consideration and discussion of the data. We believe that this new data is significantly important in the design of high-performance autonomous molecular robots.

## 2. Experimental Section

### 2.1. Synthesis of poly(NIPAAm-co-Ru(bpy)_3_-co-AMPS)

**Figure 1.**
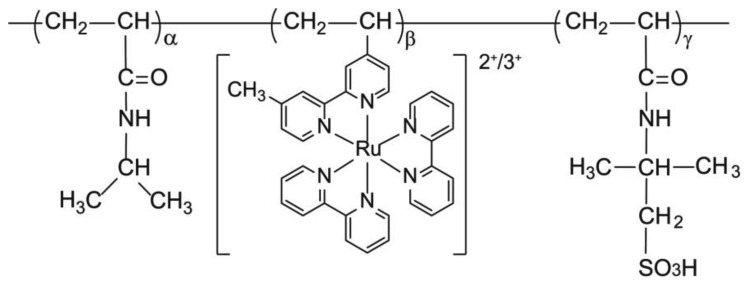
Chemical structure of poly(NIPAAm-*co*-Ru(bpy)_3_-*co*-AMPS).

Poly(NIPAAm-*co*-Ru(bpy)_3_-*co*-AMPS) (Figure 1) was synthesized by radical polymerization using NIPAAm, AMPS, and Ru(bpy)_3_ monomers, and 2,2′ -azobisisobutyronitrile (AIBN) as an initiator, in a mixture of ethanol and water (1:1 wt/wt%), with a total monomer concentration of 20 wt% at 60 °C. The feed composition was NIPAAm:Ru(bpy)_3_:AMPS = 40:10:50 (wt%). The resulting reaction mixture was dialyzed against water for 4 d, followed by ethanol for 3 d, and then freeze-dried.

### 2.2. Synthesis of poly(NIPAAm-co-Ru(bpy)_3_)

**Figure 2.**
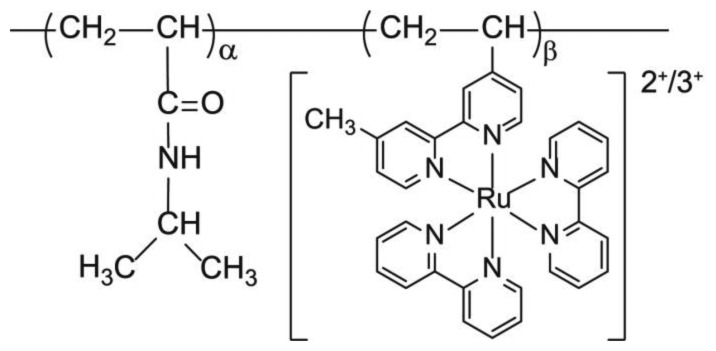
Chemical structure of poly(NIPAAm-*co*-Ru(bpy)_3_).

Poly(NIPAAm-*co*-Ru(bpy)_3_) (Figure 2) was synthesized by radical polymerization using NIPAAm and Ru(bpy)_3_ monomers, and AIBN in ethanol, with a total monomer concentration of 20 wt%, at 60 °C. The feed composition was NIPAAm:Ru(bpy)_3_ = 10:90 (wt%). The resulting reaction mixture was dialyzed against water for 4 d, followed by ethanol for 3 d, and then freeze-dried.

### 2.3. Measurement of Lower Critical Solution Temperature (LCST)

The LCSTs of the poly(NIPAAm-*co*-Ru(bpy)_3_-*co*-AMPS) and poly(NIPAAm-*co*-Ru(bpy)_3_) solutions in the reduced and oxidized states were measured by using oxidizing and reduced agents, respectively. The 0.5 wt% polymer solutions in the reduced and oxidized states were prepared by dissolving the polymer in a 0.3 M HNO_3_ aqueous solutions including the 5 mM Ce(SO_4_)_2_ or 5 mM Ce_2_(SO_4_)_3_, respectively. The LCST measurements for the polymer solutions were conducted with a spectrophotometer (Model V-630, JASCO, Tokyo, Japan) equipped with magnetic stirrers and a thermostatic controller. The LCST measurement was carried out by using the 570 nm wavelength because of the isosbestic point for the polymer solutions in the reduced and oxidized states. The change in the transmittance (%) for the polymer solutions were measured by raising the temperature at a rate of 0.5 °C/min.

### 2.4. Measurement of Transmittance of Self-Oscillations

The self-oscillating polymer solutions were prepared by dissolving the polymer (0.5 wt%) in an aqueous solution containing the three BZ substrates, *i.e.*, nitric acid (HNO_3_), sodium bromate (NaBrO_3_), and malonic acid (MA). The transmittance of the self-oscillations of the polymer solutions were measured at a constant temperature (18 °C) with stirring. A wavelength of 570 nm was used to detect the changes in transmittance, which are based on the autonomous aggregation-disaggregation of the polymers. This is because 570 nm is the isosbestic point of the reduced and oxidized states of the Ru(bpy)_3_ moiety in the polymer chain [21,28]. The time course of the transmittance at 570 nm was monitored using a spectrophotometer (JASCO Model V-630).

## 3. Results and Discussion

**Figure 3.**
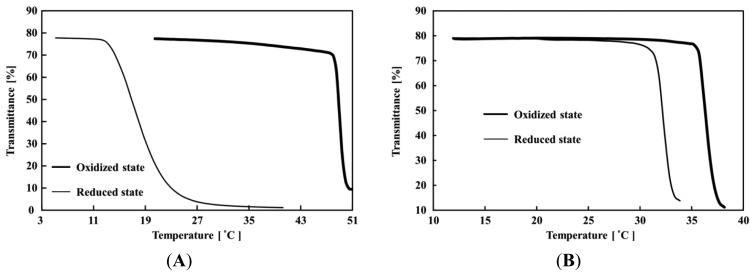
Relationship between transmittance and temperature for the poly(NIPAAm-*co*-Ru(bpy)_3_-*co*-AMPS) (**A**) and the poly(NIPAAm-*co*-Ru(bpy)_3_) (**B**).

Figure 3 shows the relationship between the transmittance and temperature for the poly(NIPAAm-*co*-Ru(bpy)_3_-*co*-AMPS) and poly(NIPAAm-*co*-Ru(bpy)_3_) solutions. The LCSTs of the poly(NIPAAm-*co*-Ru(bpy)_3_-*co*-AMPS) in the reduced and oxidized states were 13.5 °C and 48 °C, respectively. In contrast, for the poly(NIPAAm-*co*-Ru(bpy)_3_), the LCSTs in the reduced and oxidized states were 31.5 °C and 36 °C, respectively. The LCST of the poly(NIPAAm) solution was 31 °C [32]. Therefore, the LCST of the poly(NIPAAm-*co*-Ru(bpy)_3_) in the oxidized state was slightly higher than that of poly(NIPAAm) solution. In the reduced state, the LCST was almost the same as that of the poly(NIPAAm) solution. This result demonstrated that the solubility of the Ru(bpy)_3_^3+^ moiety in the polymer chain was higher than that of Ru(bpy)_3_^2+^. This difference in solubility was the origin of the optical self-oscillation of the polymer chain. In the case of the AMPS-containing polymer solution, the LCSTs in the reduced and oxidized states were significantly different from the poly(NIPAAm-*co*-Ru(bpy)_3_) solution. In the reduced state, the LCST of the AMPS-containing polymer solution decreased 18 °C as compared to the poly(NIPAAm-*co*-Ru(bpy)_3_) solution. This lower LCST was attributed to the interaction between the negatively charged AMPS domain and the reduced Ru(bpy)_3_^2+^ moiety. That is, the AMPS domain in the polymer chain interacted with the Ru(bpy)_3_^2+^ moiety among the polymer chains, and the polymer chains aggregated at low temperatures. On the other hand, in the oxidized state, the LCST increased 12 °C as compared to the poly(NIPAAm-*co*-Ru(bpy)_3_) solution. Generally, the LCST of the NIPAAm with the charged domain increased due to the increase in the hydrophilicity of the polymer chain. Therefore, the AMPS domain and Ru(bpy)_3_^3+^ hardly interacted as compared to the interaction with Ru(bpy)_3_^2+^, and the AMPS moiety increased the LCST for the same reason as the NIPAAM with the charged domain.

**Figure 4.**
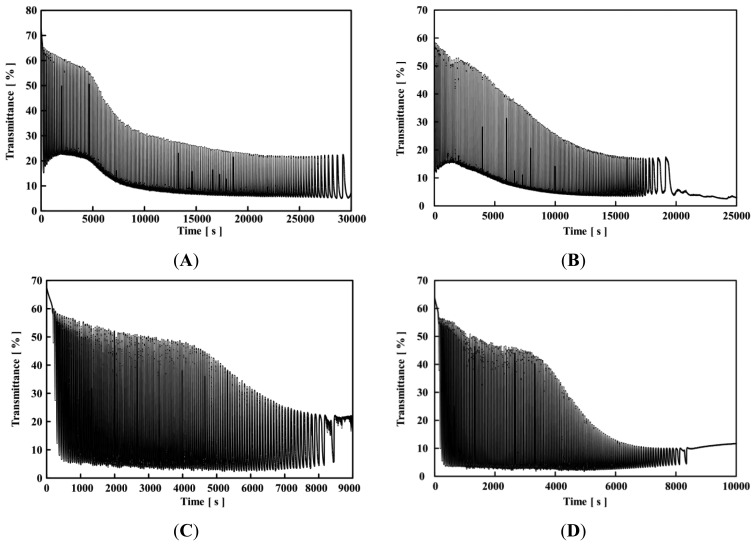
Oscillating profiles of transmittance at 18 °C for 0.5 wt% poly(NIPAAm-*co*-Ru(bpy)_3_-*co*-AMPS) solutions with fixed concentrations of HNO_3_ and MA ([HNO_3_] = 0.3 M and [MA] = 0.1 M; (**A**) [NaBrO_3_] = 0.2 M; (**B**) [NaBrO_3_] = 0.3 M; (**C**) [NaBrO_3_] = 0.7 M; and (**D**) [NaBrO_3_] = 0.8 M).

**Figure 5.**
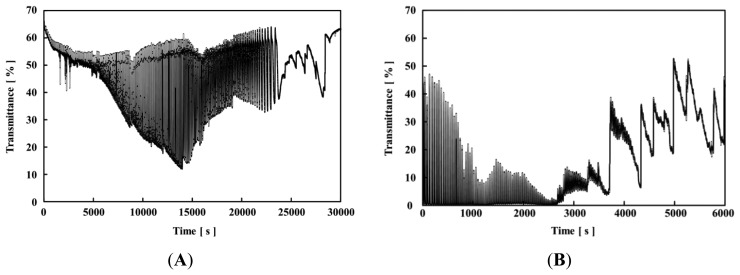
Oscillating profiles of transmittance at 18 °C for poly(NIPAAm-*co*-Ru(bpy)_3_) solutions with fixed HNO_3_ and MA concentrations ([HNO_3_] = 0.3 M and [MA] = 0.1 M; (**A**) [NaBrO_3_] = 0.3 M; (**B**) [NaBrO_3_] = 0.7 M).

Figures 4 and 5 show the self-oscillating behaviors of the poly(NIPAAm-*co*-Ru(bpy)_3_-*co*-AMPS) and poly(NIPAAm-*co*-Ru(bpy)_3_) solutions, respectively, with different concentrations of sodium bromate at 18 °C, and fixed concentrations of MA ([MA] = 0.1 M) and HNO_3_ ([HNO_3_] = 0.3 M). As shown in Figures 4 and 5, the self-oscillation of the polymer chains was induced by the BZ reaction. The autonomous color change of the polymer solution in the reduced and oxidized states could not be detected by the transmittance of the self-oscillation because the optical measurement utilized the wavelength of 570 nm, the isosbestic point of the reduced and oxidized states of the Ru(bpy)_3_ moiety in the polymer chain. In order to induce the autonomous self-oscillation of the polymer chains by the BZ reaction, the polymer solution and solutions of the three BZ substrates were mixed just before starting the transmittance measurements. Generally, as the ionic strength increases, the solubility of the charged polymer chain decreases. Here, the ionic strength of the polymer solution was high because the BZ reaction requires high concentrations of the BZ substrates. As shown in Figure 4, at the beginning of the self-oscillation, the transmittance values in the reduced state were low, that is, the baseline of the self-oscillation consisted of a low transmittance value. The transmittance value is determined by the size of the polymer aggregation. Therefore, the size of the polymer aggregate in the reduced state increased as a result of the decrease in the solubility due to the high ionic strength of the solution.

During the self-oscillation, when the Ru(bpy)_3_ moiety in the polymer chain changed from the reduced state to the oxidized state, the transmittance value rapidly increased because the solubility of the polymer chain increased as shown in Figure 3A. This result demonstrated that the large amount of aggregated polymers in the reduced state disaggregated the small amount of aggregated polymers in the oxidized state, due to the change in the hydrophilicity of the polymer chain induced by the BZ reaction. Moreover, as the self-oscillation advanced, the amplitude of the self-oscillation decreased with time, *i.e.*, the self-oscillation exhibited damping. The mechanism of the damping can be explained as follows: In the BZ reaction, the time in the reduced state is much longer than that in the oxidized state. Therefore, the low solubility of the polymer chain in the reduced state determined the polymer aggregation state in the self-oscillating behavior. As the aggregation-disaggregation self-oscillation repeated, the size of the polymer aggregate in the reduced state increased because of the hydrophobicity of the polymer chain. Therefore, once the attenuation of the transmittance of the self-oscillation begins, the amplitude of the self-oscillation never recovered because the polymer aggregation state is thermodynamically more stable in the polymer solution. Finally, the self-oscillation completely terminated.

In addition, as shown in Figure 4A,B, the baseline of the self-oscillation increased, and gradually decreased. The polymer solution and solutions of the three BZ substrates (HNO_3_, MA, and NaBrO_3_) were mixed immediately before the measurement. In Figure 4A,B, when the ionic strength rapidly increased, the polymer chain formed excessively large polymer aggregates. This phenomenon can be observed in many types of polymer chains. Therefore, the excessively large polymer aggregates disaggregated with time, and the baseline of the self-oscillation increased gradually. Subsequently, the disaggregated polymer chain in the reduced state re-aggregated because of the strong hydrophobicity of the polymer chain with the reduced Ru(bpy)_3_. That is, in the beginning of the self-oscillation in Figure 4A,B, the low baseline was due to the ionic strength of the solution, and subsequently, the decreasing baseline was attributed to the aggregation of the polymer chain with the reduced Ru(bpy)_3_ moiety. In Figure 4C,D, the baseline of the self-oscillation was significantly small from the beginning of the self-oscillation due to the high ionic strength, and the aggregation of the polymer chains. The polymer chains were highly aggregated so the baseline did not increase.

Furthermore, the lifetime of the self-oscillation decreased with an increase in the concentration of the sodium bromate. In the case of [NaBrO_3_] = 0.2 and 0.3 M, the self-oscillation time was more than 20,000 s. In contrast, when [NaBrO_3_] = 0.6 and 0.7 M, the life time of the self-oscillation was about 8,000 s. As the concentration of NaBrO_3_ increased, the ionic strength of the polymer solution increased. Therefore, with a high concentration of NaBrO_3_ and a shorter amount of time, the large polymer aggregates hardly dissociated even in the oxidized state. Therefore, the self-oscillation stopped a shorter amount of times as compared to experiment utilizing a low concentration of NaBrO_3_.

As for the poly(NIPAAm-*co*-Ru(bpy)_3_) solution, when [NaBrO_3_] = 0.3 M (Figure 5A), at the beginning of self-oscillation (about 0–5,000 s), the base line of the self-oscillation was significantly higher than that of the AMPS-containing polymer solutions. This result demonstrated that the effect of ionic strength for poly(NIPAAm-*co*-Ru(bpy)_3_) was smaller than that for the poly(NIPAAm-*co*-Ru(bpy)_3_-*co*-AMPS) because the AMPS-containing polymer chain has negatively charged AMPS moieties (40 mol%). In contrast, when [NaBrO_3_] = 0.7 M (Figure 5B), the beginning of the self-oscillating behavior (about 0–3,000 s) resembled that of poly(NIPAAm-*co*-Ru(bpy)_3_-*co*-AMPS) (see Figure 4). This result demonstrated that the high ionic strength caused the aggregation of poly(NIPAAm-*co*-Ru(bpy)_3_) in the beginning of the self-oscillation.

As shown in Figure 5A, when the self-oscillation was repeated, the base line of the self-oscillation gradually decreased because the size of the polymer aggregate in the reduced state increased with time. After decreasing the baseline, the transmittance value of poly(NIPAAm-*co*-Ru(bpy)_3_) increased again. In a previous study, this behavior was observed using self-oscillating polymer chains with positively charged methacrylamidopropyltrimethylammonium chloride (MAPTAC) [33]. In the transmittance of the self-oscillation of the MAPTAC-containing polymer solution, the initially decreased transmittance value increases again. This phenomenon originates in the autonomous dissociation of the large polymer aggregates as a result of the electrostatic repulsive force of the MAPTAC component. In the case of the poly(NIPAAm-*co*-Ru(bpy)_3_-*co*-AMPS) solution, once the transmittance of the self-oscillation began to attenuate, the decreased amplitude never recovers. The AMPS-containing polymer chain had both anionic and cationic moieties. Therefore, the inter- and intra-electrostatic interactions among the polymer chains led to the polymer aggregation, and the large polymer aggregates never disaggregated during the self-oscillation. In contrast, the MAPTAC-containing polymer chain only had a cationic moiety. This difference in the charged state of the polymer chain is attributed to the differences in the self-oscillating behaviors of the polymers. The poly(NIPAAm-*co*-Ru(bpy)_3_) only had a positive charge. Therefore, the repulsive force of the positively charged moiety encouraged the disaggregation. As shown in Figure 5A, the baseline of the self-oscillation increased again after about 15,000 s due to the dissociation of the polymer aggregates in the reduced state. In the case of Figure 5B, after about 3,000 s, the baseline of the self-oscillation increased due to the dissociation of the polymer aggregates. In addition, the lifetime of the self-oscillation for the poly(NIPAAm-*co*-Ru(bpy)_3_) solution decreased with an increase in the concentration of NaBrO_3_. The ionic strength of the solutions had a significantly effect on the solubility of the polymer chain. The transmittance value of the baseline for poly(NIPAAm-*co*-Ru(bpy)_3_) was different when [NaBrO_3_] = 0.3M and [NaBrO_3_] = 0.7 M in the same fashion with the results obtained with the AMPS-containing polymer solution. Therefore, the lifetime of the poly(NIPAAm-*co*-Ru(bpy)_3_) solution was affected by the concentration of NaBrO_3_ in the same manner as poly(NIPAAm-*co*-Ru(bpy)_3_-*co*-AMPS).

**Figure 6.**
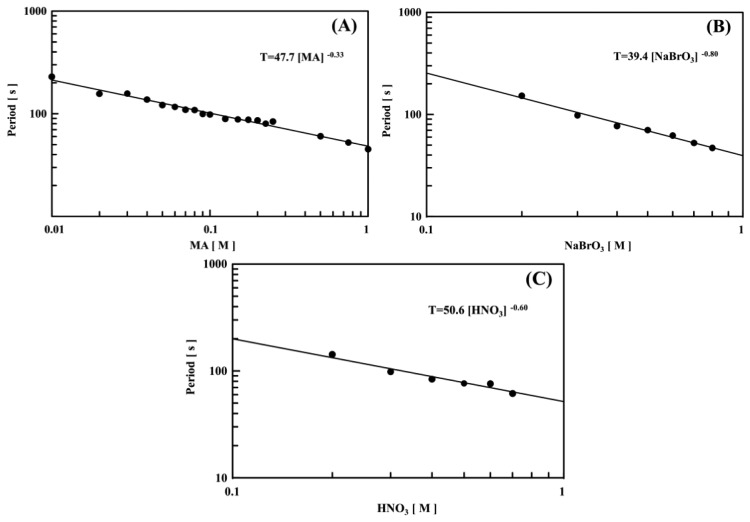
Logarithmic plots of period *T* (s) for 0.5 wt% poly(NIPAAm-*co*-Ru(bpy)_3_-*co*-AMPS) solution vs. initial molar concentration of one BZ substrate at constant temperature (*T* = 18 °C), with fixed concentrations of the other two BZ substrates: (**A**) [NaBrO_3_] = 0.3 M and [HNO_3_] = 0.3 M; (**B**) [MA] = 0.1 M and [HNO_3_] = 0.3 M; and (**C**) [MA] = 0.1 M and [NaBrO_3_] = 0.3 M.

**Figure 7.**
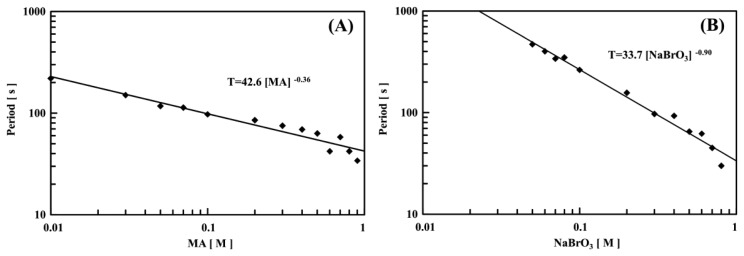

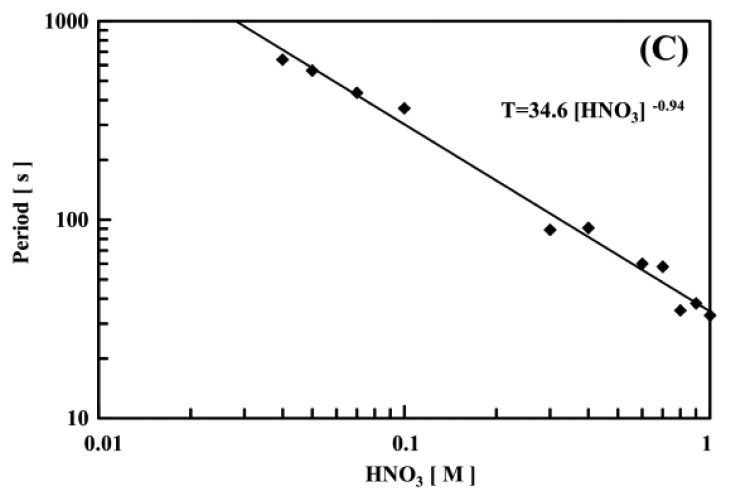
Logarithmic plots of period *T* (s) of 0.5 wt% poly(NIPAAm-*co*-Ru(bpy)_3_) solution vs. initial molar concentration of one BZ substrate at constant temperature (*T* = 18 °C), with fixed concentrations of the other two BZ substrates: (**A**) [NaBrO_3_] = 0.3 M and [HNO_3_] = 0.3 M; (**B**) [MA] = 0.1 M and [HNO_3_] = 0.3 M; and (**C**) [MA] = 0.1 M and [NaBrO_3_] = 0.3 M.

Figures 6 and 7 show logarithmic plots of the period against the initial concentration of one substrate, with fixed concentrations of the other two BZ substrates at a constant temperature (*T* = 18 °C). As shown in Figures 6 and 7, all the logarithmic plots exhibited good linear relationships. The period, *T* (s), of the self-oscillation can be expressed as *a*[substrate]*^b^*, where *a* and *b* are experimental constants and the brackets denote initial concentration. When the concentrations of NaBrO_3_ and MA were altered, the *b* values were almost the same as compared to the two types of the polymer chains. In contrast, as shown in Figures 6C and 7C, the *b* value of poly(NIPAAm-*co*-Ru(bpy)_3_) was smaller than that of poly(NIPAAm-*co*-Ru(bpy)_3_-*co*-AMPS). This result indicated that the concentration of H^+^ could increase the period of the optical self-oscillation for the poly(NIPAAm-*co*-Ru(bpy)_3_) as compared to the poly(NIPAAm-*co*-Ru(bpy)_3_-*co*-AMPS). We considered that this effect was related to the strongly acidic AMPS domain in the self-oscillating polymer chain. In addition, as shown in Figures 6C and 7C, the periods have different characteristics as compared with those of the conventional poly(NIPAAm-co-Ru(bpy)_3_) gels [34]. In the case of a poly[NIPAAm-co-Ru(bpy)_3_] gel, the period increases with increasing HNO_3_ concentration. In general, the self-oscillation period decreases with increased initial concentrations of the BZ substrates because of the increase in the collision frequency among the BZ substrates. Under these experimental conditions, therefore, as the initial concentration of nitric acid increased, the periods of the two types of polymer chains decreased.

**Figure 8.**
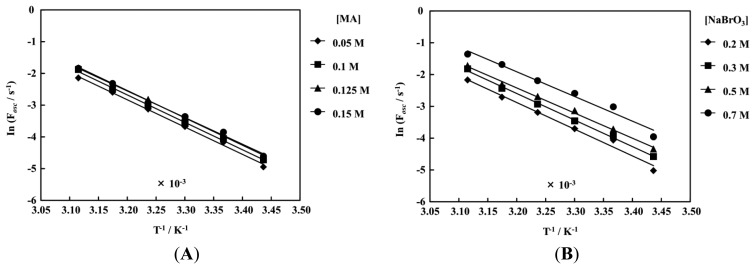

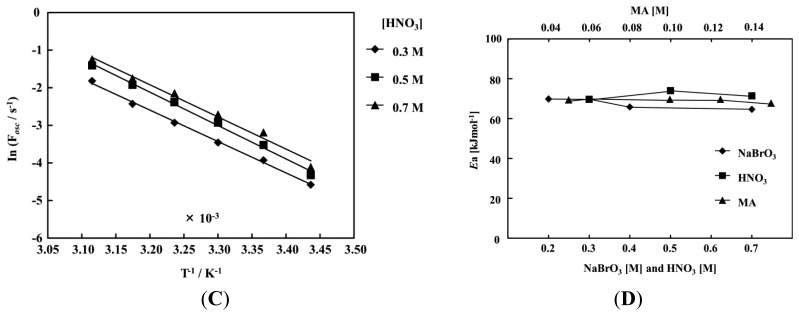
Arrhenius dependence on temperature of self-oscillation for the poly(NIPAAm-*co*-Ru(bpy)_3_-*co*-AMPS), oscillating frequency (F*osc*), with a fixed concentration of the other two BZ substrates: (**A**) [NaBrO_3_] = 0.3 M and [HNO_3_] = 0.3 M; (**B**) [MA] = 0.1 M and [HNO_3_] = 0.3 M; (**C**) [NaBrO_3_] = 0.3 M and [MA] = 0.1 M; (**D**) Dependence of the activation energy on concentration of the three BZ substrates.

**Figure 9.**
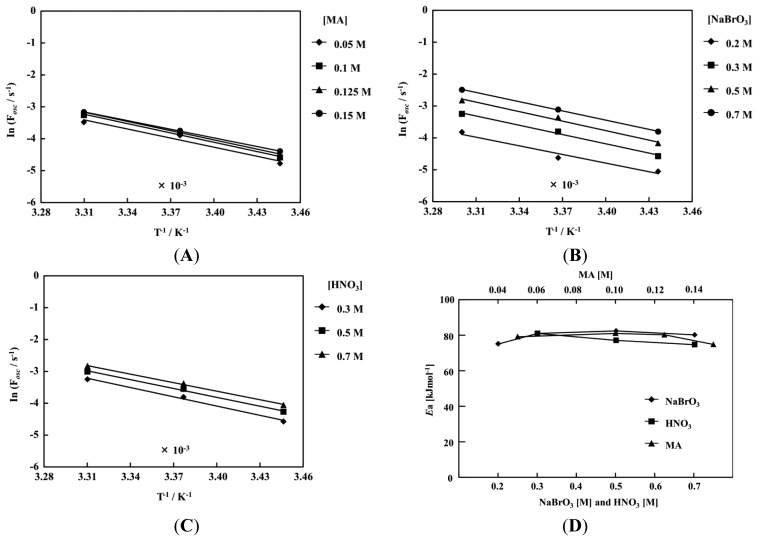
Arrhenius dependence on temperature of the self-oscillation of the poly(NIPAAm-*co*-Ru(bpy)_3_), F*osc*, with fixed concentrations of the other two BZ substrates: (**A**) [NaBrO_3_] = 0.3 M and [HNO_3_] = 0.3 M; (**B**) [MA] = 0.1 M and [HNO_3_] = 0.3 M; (**C**) [NaBrO_3_] = 0.3 M and [MA] = 0.1 M; (**D**) Dependence of activation energy on concentration of the three BZ substrates.

Figures 8 and 9 show the Arrhenius dependence on the temperature at a fixed concentration of the other two BZ substrates. The measurements of the poly(NIPAAm-*co*-Ru(bpy)_3_-*co*-AMPS) and poly(NIPAAm-*co*-Ru(bpy)_3_) were conducted in the temperature ranges of 18–48 °C and 18–30 °C, respectively. In the case of the poly(NIPAAm-*co*-Ru(bpy)_3_), the self-oscillating behavior could not be measured at 36 °C above. This is because the LCST in the oxidized state was 36 °C. Therefore, above 36 °C the poly(NIPAAm-*co*-Ru(bpy)_3_) aggregated in the reduced and oxidized state, and the self-oscillation did not occur. As shown in Figures 8 and 9, all plots have a linear relationship, that is, the effect of the temperature on the period followed the same trend. In addition, it was clarified that the activation energies of the self-oscillation was hardly affected by the initial concentration of the three BZ substrates as shown in Figures 8D and 9D. Moreover, activation energies of the two self-oscillating polymer chains were almost the same value as the normal BZ reaction, *i.e.*, not including the self-oscillating polymer system with the Ru moiety [35]. These results suggested that the polymer chain covalently bonded to the Ru(bpy)_3_ did not inhibit the reaction when poly(NIPAAm-*co*-Ru(bpy)_3_-*co*-AMPS) and poly(NIPAAm-*co*-Ru(bpy)_3_) were utilized.

## 4. Conclusions

In this study, we investigated the self-oscillating behaviors of two types of polymer chains. The LCSTs of the two types of the self-oscillating polymer chains were significantly different owing to the effect of the negatively charged AMPS domain. This significant difference in solubility could be attributed to the different behavior in the self-oscillation. It was demonstrated that the self-oscillating behavior (waveform and life time) was significantly affected by the ionic strength of the polymer solution. In addition, it was clarified that the period of the self-oscillation could be controlled by the initial concentration of the three BZ substrates and the temperature. Moreover, it was demonstrated that the activation energies of the self-oscillation was hardly affected by the initial concentration of the three BZ substrates. The activation energies of the two types of the polymer chains were almost the same as the normal BZ reaction, *i.e.*, not including the self-oscillating polymer system with the Ru moiety. These results clarified that the polymer chain covalently bonded to Ru(bpy)_3_ does not inhibit the reaction when poly(NIPAAm-*co*-Ru(bpy)_3_-*co*-AMPS) and poly(NIPAAm-*co*-Ru(bpy)_3_) are utilized.

## References

[1] Badmkc J.D., Balzani V., Credi A., Silvi S., Stoddart J.F. (2004). A molecular elevator. Science.

[2] Soong R.K., Bzchand G.D., Neves H.P., Olkhovets A.G., Craighead H.G., Montemagno C.D. (2000). Powering an inorganic nanodevice with a biomolecular motor. Science.

[3] Hugel T., Holland N.B., Cattani A., Moroder L., Seitz M., Gaub H.E. (2002). Single-molecule optomechanical cycle. Science.

[4] Ito Y., Park Y.S., Imanishi Y. (1997). pH-Sensitive gating by conformational change of a polypeptide brush grafted onto a porous polymer membrane. J. Am. Chem. Soc..

[5] Seeman N.C. (1982). Nucleic acid junctions and lattices. J. Theor. Biol..

[6] Winfree E., Liu F., Wenzler L., Seeman N. (1998). Design and self-assembly of two-dimensional DNA crystals. Nature.

[7] Yan H., Park S.H., Finkelstein G., Reif J., LaBean T. (2003). DNA-templated self-assembly of protein arrays and highly conductive nanowires. Science.

[8] He Y., Chen Y., Liu H., Ribbe A., Mao C. (2005). Self-assembly of hexagonal DNA two-dimensional (2D) Arrays. J. Am. Chem. Soc..

[9] Hamada S., Murata S. (2009). Substrate-assisted assembly of interconnected single-duplex DNA nanostructures. Angew. Chem. Int. Ed..

[10] Kuzuya Y., Sakai T., Yamazaki Y., Xu Y., Komiyama M. (2011). Nanomechanical DNA origami ‘single-molecule beacons’ directly imaged by atomic force microscopy. Nat. Commun..

[11] Murata S., Konagaya A., Kobayashi S., Saito H., Hagiya M. (2013). Molecular robotics: A new paradigm for artifacts. New Generat. Comput..

[12] Beebe D.J., Moore J.S., Bauer J.M., Yu Q., Liu R.H., Devadoss C., Jo B.H. (2000). Functional hydrogel structures for autonomous flow control inside microfluidic channels. Nature.

[13] Mukai K., Asaka K., Sugino T., Kiyohara K., Takeuchi I., Terasawa N., Futaba D.N., Hata K., Fukushima T., Aida T. (2009). Highly conductive sheets from millimeter-long single-walled carbon nanotubes and ionic liquids: Application to fast-moving, low-voltage electromechanical actuators operable in air. Adv. Mater..

[14] Tanaka T. (1981). Gels. Sci. Am..

[15] Dong L, Agarwal1 K.A., Beebe D.J., Jiang H. (2006). Adaptive liquid microlenses activated by stimuli-responsive hydrogels. Nature.

[16] Sidorenko A., Krupenkin T., Taylor A., Fratzl P., Aizenberg J. (2007). Reversible switching of hydrogel-actuated nanostructures into complex micropatterns. Science.

[17] Asoh T., Matsusaki M., Kaneko T., Akashi M. (2008). Fabrication of temperature-responsive bending hydrogels with a nanostructured gradient. Adv. Mater..

[18] Ma M., Guo L., Anderson D.G., Langer R. (2013). Bio-inspired polymer composite actuator and generator driven by water gradients. Science.

[19] Ishiwatari T., Kawaguchi M., Mitsuishi M. (1984). Oscillatry reactions in polymer systems. J. Polym. Sci. Polym. Chem..

[20] Yoshida R., Takahashi T., Yamaguchi T., Ichijo H. (1996). Self-oscillating gel. J. Am. Chem. Soc..

[21] Yoshida R., Sakai T., Ito S., Yamaguchi T. (2002). Self-oscillation of polymer chains with rhythmical soluble-insoluble changes. J. Am. Chem. Sci..

[22] Zaikin A.N., Zhabotinsky A.M. (1970). Concentration wave propagation in two-dimensional liquid-phase self-oscillating system. Nature.

[23] Reusser E.J., Field R.J. (1979). The transition from phase waves to trigger waves in a model of the Zhabotinskii reaction. J. Am. Chem. Soc..

[24] Scott S.K. (1991). Chemical Chaos.

[25] Field R.J., Burger M. (1985). Oscillations and Traveling Waves in Chemical Systems.

[26] Nicolis G., Prigogine I. (1977). Self Orgainization in Nonequilibrium Systems.

[27] Murray J.D. (1990). Mathematical Biology.

[28] Hara Y., Yoshida R. (2005). Self-oscillation of polymer chains induced by the Belousov-Zhabotinsky reaction under acid-free conditions. J. Phys. Chem. B.

[29] Hara Y., Yoshida R. (2008). A viscosity self-oscillation of polymer solution induced by the BZ reaction under acid-free condition. J. Chem. Phys..

[30] Hara Y., Yoshida R. (2005). Control of oscillating behavior for the self-oscillating polymer with pH-control site. Langmuir.

[31] Hara Y. Autonomous Polymer Actuators.

[32] Heskins M., Guillet J.E. (1968). Solution properties of Poly(N-isopropylacrylamide). J. Macromol. Sci. Chem..

[33] Hara Y., Yoshida R. (2009). Influence of a positively charged moiety on aggregation–disaggregation self-oscillation induced by the BZ reaction. Macromol. Chem. Phys..

[34] Yoshida R., Tanaka M., Onodera S., Yamaguchi T., Kokufuta E. (2000). In-phase synchronization of chemical and mechanical oscillations in self-oscillating gels. J. Phys. Chem. A.

[35] Kuhnert L., Krug H.J. (1987). Kinetics of chemical waves in the acidic bromate-malonic acid-tris(bipyridine)ruthenium(2+) system in comparison with the ferroin system. J. Phys. Chem..

